# Supplementary material for: Association between oral microbiome and seven types of cancers in East Asian population: a two-sample Mendelian randomization analysis
Source: Front Mol Biosci. 2023 Nov 21;10:1327893. doi: 10.3389/fmolb.2023.1327893 (PMC10702768; doi:10.3389/fmolb.2023.1327893)
Supplement: Supplementary file 1 [file DataSheet1.ZIP › Supplementary Materials/MR plots for tongue/tongue═╝/Prostate cancer/pheno.1506_to_prostate cancer_leave_one_out.pdf]

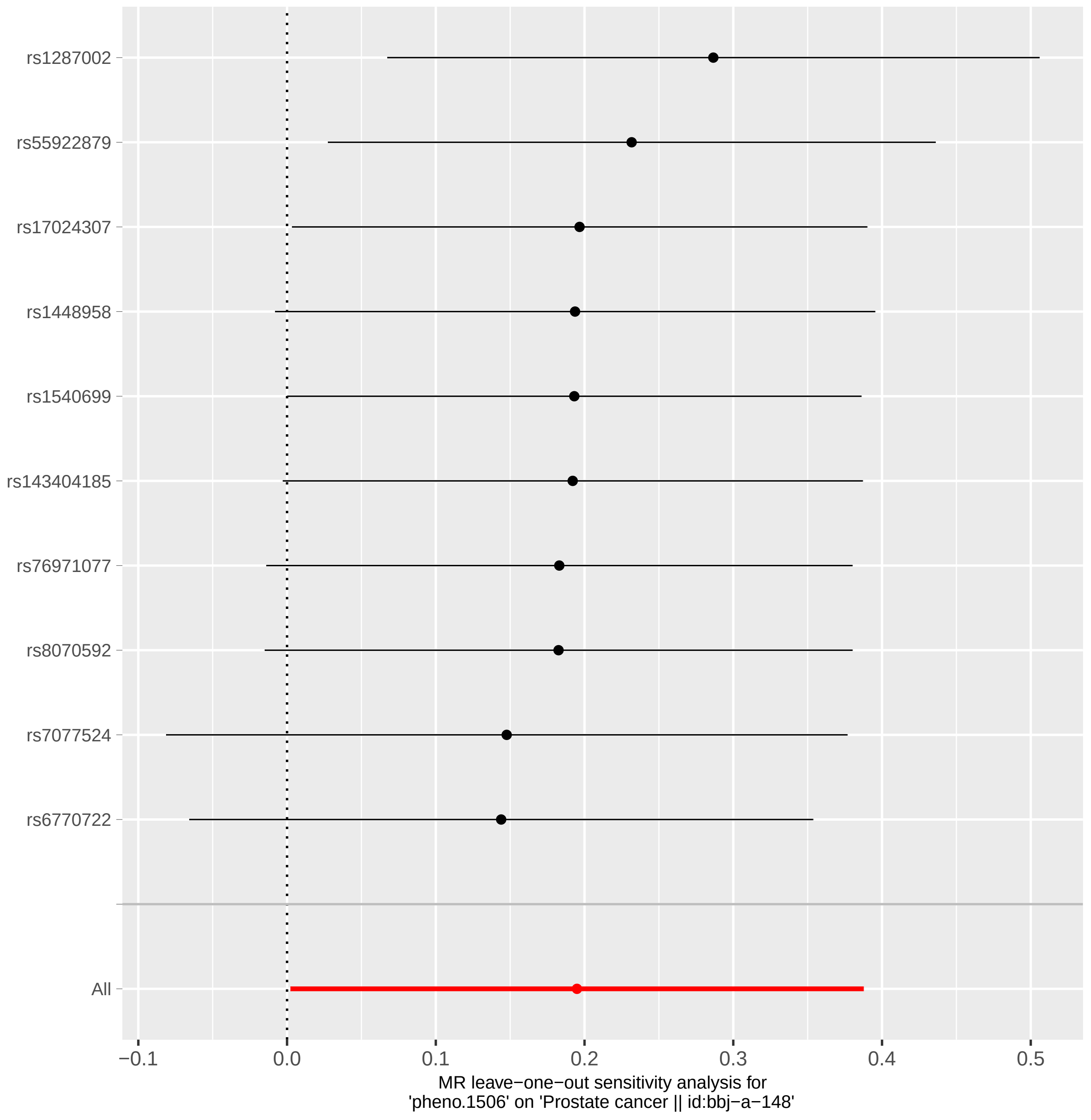
rs1287002
rs55922879
rs17024307
rs1448958
rs1540699
rs143404185
rs76971077
rs8070592
rs7077524
rs6770722
All
−0.1
0.0
0.1
0.2
0.3
0.4
0.5
MR leave−one−out sensitivity analysis for
'pheno.1506' on 'Prostate cancer || id:bbj−a−148'
